# Viscosity Deviation Modeling for Binary and Ternary Mixtures of Benzyl Alcohol-N-Hexanol-Water

**DOI:** 10.3390/ma15165699

**Published:** 2022-08-18

**Authors:** Iuliana Bîrgăuanu, Maricel Danu, Cătălin Lisa, Florin Leon, Silvia Curteanu, Constanta Ibanescu, Gabriela Lisa

**Affiliations:** 1 Faculty of Chemical Engineering and Environmental Protection “Cristofor Simionescu”, Gheorghe Asachi Technical University, 73 Prof. Dr. Doc. D. Mangeron Street, 700050 Iasi, Romania; 2 Petru Poni Institute of Macromolecular Chemistry of Iaşi, 41A Aleea Gr. Ghica Voda, 700487 Iasi, Romania; 3 Faculty of Automatic Control and Computer Engineering, Gheorghe Asachi Technical University, 27A Prof. Dr. Doc. D. Mangeron Street, 700050 Iasi, Romania

**Keywords:** viscosity deviation modeling, ANN, benzyl alcohol, n-hexanol, water

## Abstract

Knowing the thermodynamic and transport properties of liquid systems is very important in engineering for the development of theoretical models and for the design of new technologies. Models that allow accurate predictions of thermodynamic and transport properties are needed in chemical engineering calculations involving fluid, heat, and mass transfer. In this study, the modeling of viscosity deviation for binary and ternary systems containing benzyl alcohol, n-hexanol, and water, less studied in the literature, was carried out using Redlich and Kister (R-L) models, multiple linear regression (MLR) models and artificial neural networks (ANN). The viscosity of the binary and ternary systems was experimentally determined at the following temperatures: 293.15, 303.15, 313.15, and 323.15 K. Viscosity deviation was calculated and then correlated with mole fractions, normalized temperature, and refractive index. The neural model that led to the best performance in the testing and validation stages contains 4 neurons in the input layer, 12 neurons in the hidden layer, and one neuron in the output layer. In the testing stage for this model, the standard deviation is 0.0067, and the correlation coefficient is 0.999. In the validation stage, a deviation of 0.0226 and a correlation coefficient of 0.996 were obtained. The MLR model led to worse results than those obtained with the neural model and also with the R-L models. The standard deviation for this model is 0.099, and the correlation coefficient is 0.898. Its advantage over the R-L type models is that the influence of both composition and temperature are included in a single equation.

## 1. Introduction

Many products from the pharmaceutical or cosmetic industries consist of liquid systems in the form of solutions that contain more than one solute. After they are used, they reach the environment and can negatively influence it. Environmental pollution with pharmaceuticals and cosmetics has represented a particularly important global problem since ancient times [1]. The presence of pharmaceutical and cosmetic products in surface waters negatively affects living organisms. Also, if they reach the human body, they can cause various ailments. In order to reduce these negative effects on the environment, numerous groups of researchers from around the world have focused their attention on studies on the behavior of liquid mixtures [2,3,4,5,6,7,8,9,10,11,12,13,14]. It can be said that the pollution caused by the products of the pharmaceutical and cosmetic industry represents an ever-increasing challenge for the protection of the environment because the human population registers a systematic increase in the use of medicines and personal care products [2]. Emerging pollutants reach the environment and pollute wastewater through various pathways. Medicines and pharmaceutical skin products such as creams, ointments, and body lotions belong to the category of emerging pollutants. Since they are not completely absorbed by the skin, they can contaminate surface waters. Other examples of emerging pollutants can be personal care products or some soaps. Benzyl alcohol and n-hexanol are the most widely applied solvents in the pharmaceutical and cosmetic industries.

The properties most frequently evaluated in the case of aqueous solutions are density, viscosity, refractive index, surface tension, thermal conductivity, etc. The importance of evaluating the thermodynamic and transport properties of aqueous solutions derives from the need to know them for the development of theoretical models and for the design and development of new technologies. Knowing the dynamic viscosity of liquid mixtures is imperative for chemical engineering calculations involving fluid, heat, and mass transfer [3]. According to the literature, the viscosity data of liquid mixtures for binary systems containing n-hexanol and ethanol were reported by Cano-Gómez et al. They highlighted negative viscosity deviations from a mole fraction weighted average of the pure component viscosities over the entire composition range [4]. Indraswati and his collaborators determined the viscosity for binary mixtures containing n-hexanol and ethyl valerate or hexyl acetate, also obtaining negative viscosity deviations over the entire composition range [5]. Other data for the viscosity of binary systems containing n-hexanol were reported by Audonnet et al. [6], Domanska et al. [7], Živković et al. [8], Estrada-Baltazar et al. [9], and Das et al. [10]. In all these studies, the viscosity deviation was correlated with the composition with polynomial models of the Redlich and Kister type. This model correlates the excess properties of a multicomponent system with the mole fractions of the components in the mixture for each temperature. The advantages of the Redlich and Kister type models are given by the simplicity of the equations used and the very good performances obtained.

A significant contribution to the study of the transport properties for benzyl alcohol in different binary and ternary systems was made by Cruz and his collaborators and obtained for the ternary system (benzaldehyde-toluene-benzyl alcohol), a standard deviation between the experimental viscosities and those calculated with a Redlich and Kister type model of 1.1% [11]. Nayak et al. evaluated the viscosity of benzyl alcohol in the binary mixture formed by ethyl chloroacetate—benzyl alcohol at the following temperature values: 298.15, 303.15, and 308.15 K. The obtained results were correlated with a polynomial model of the Redlich–Kister type [12]. Gramajo De Doz et al. calculated the viscosity deviation for the ternary system benzyl alcohol, 4-hydroxy-4-methyl-2-pentanone, and water and correlated the obtained values with mole fractions also using the Redlich–Kister model [13]. Chen et al. determined the density, refractive index, viscosity, and surface tension for binary and ternary systems containing benzyl acetate, benzyl alcohol, and ethanol. Based on the experimental determinations, they calculated the thermodynamic properties of the excess, which were then correlated with the Redlich–Kister and Cibulkatype models. They determined that the deviation from ideal behavior is probably due to the breaking of hydrogen bonds between alcohol molecules. For Δ*η* viscosity deviations, negative values were found over the entire range of composition variation for both binary and ternary systems [14].

Studies presenting experimental determinations for the viscosity of binary or ternary systems of benzyl alcohol, n-hexanol, and water are less common in the literature.

The design and development of new water depollution technologies require very precise data on the thermodynamic properties, and in the absence of experimental data, precise predictive methods are needed. The relations between thermodynamic properties, composition, and temperature can be modeled using multiple linear regression (MLR) methods and/or various artificial intelligence tools. In particular, the study of the physicochemical properties of binary and ternary, or multicomponent liquid mixtures over a wide range of composition and temperature, represents an important source of information for establishing the relation between the internal structure of the systems and their physical properties [15].

The prediction of density, refractive index, boiling point, dielectric constant, and viscosity for a series of organic derivatives having the general structure X-CH_2_-CH_2_-Y was carried out by Cocchi et al. using MLR-type models. The performances of the obtained models were quantified by the standard deviation, finding very good correlations between the values of the properties calculated with the MLR models and those determined experimentally [16]. Zhao et al. performed the prediction of the viscosity of imidazolium-based ionic liquids using the multiple linear regression (MLR) algorithm, but also artificial intelligence tools, namely the support vector machine (SVM) algorithm. They used a consistent database. The average deviation of the entire data set was 24.2% for the MLR model and 3.95% for the SVM [17]. The statistical processing of the experimental data with the multiple linear regression (MLR) method was used by our research group to estimate some excess thermodynamic properties (the dependent variable) using the composition of the mixtures the temperature and the refractive index [18,19] as independent variables.

Artificial neural networks have been used by Mehlman et al. to make density, viscosity, and refractive index predictions for ternary and quaternary solvent mixtures using experimental determinations of the properties of binary systems [20]. Rocabruno-Valdés et al. made predictions of the dynamic density and viscosity of biodiesel from fatty acid methyl esters composition, number of carbon atoms, number of hydrogen atoms, and temperature, using single-layer neural networks with 1 to 6 hidden neurons. In the validation stage, the performance of the built models was very good, and when comparing the experimental values with the simulated ones, the correlation coefficients were higher than 0.92, and the mean squared error (MSE) was higher than 0.0018 [21]. Correlation coefficients of 0.98 and the average absolute relative deviation on the test set of 6.84% were recently obtained by Yu et al. with a multilayer perceptron neural model for predicting the viscosity of an emerging class of green solvents [22]. Other existing studies in the literature used neural networks to predict the viscosity of mixtures containing ionic liquids [23,24] or nanofluids [25]. The use of artificial neural networks for viscosity deviation modeling is less studied in the literature.

The development of models that allow predictions of the thermodynamic and transport properties of multicomponent mixtures to be made is extremely important for chemistry and chemical engineering. An important reason for these approaches is the multiple economic advantages obtained both in terms of reducing the costs of research and in the efficiency of the time needed to carry out experimental determinations, which are quite laborious, and many repetitions are necessary for accurate data.

This paper presents the experimental determination of the viscosity of binary and ternary mixtures of benzyl alcohol, n-hexanol, and water, which are less studied in the literature. Also, with the help of neural networks, models are built that correlate viscosity deviation with normalized temperature, composition, and refractive index. The obtained results are compared with those provided by Redlich and Kister type polynomial models, MLR models, and other regression algorithms.

## 2. Materials and Methods

The substances used for the experimental measurements were purchased from specialized companies, and according to the technical sheet, they have a purity level of at least 99%, namely benzyl alcohol (Purity 99.5%, Merck, Kenilworth, NJ, USA) and n-hexanol (Purity 99%, Sigma-Aldrich, St. Louis, MO, USA). Bidistilled water was used to obtain the binary and ternary systems.

The preparation of binary and ternary mixtures using the mentioned liquids was carried out by weighing on an analytical balance type XP105 from Mettler Toledo (Columbus, OH, USA), which ensures a measurement accuracy of ±0.01 mg. The estimation of mole fractions was thus achieved with a precision of ±0.0001, and this will depend on the compound used. The liquid mixtures were made in sealed vials to avoid preferential evaporation.

The viscosities of the pure components and binary and ternary mixtures were determined with a Physica MCR 501 modular rheometer equipped with a CC27 concentric cylinder measuring system at a shear rate of 10 s^−1^ and at temperatures of 293.15, 303.15, 313.15, and 323.15 K.

Based on these experimental determinations, the thermodynamic properties of the excess were evaluated, respectively the viscosity deviation (Δ*η*) was determined.

To model the viscosity deviation, a database containing information on experimental determinations of viscosity and refractive index for 27 binary and ternary mixtures containing benzyl alcohol (X_1_), n-hexanol (X_2_), and water (X_3_) was built, at four temperature values (293.15, 303.15, 313.15, and 323.15 K). Thermodynamic properties were evaluated for 6 benzyl alcohol-water binary mixtures, 9 benzyl alcohol-n-hexanol binary mixtures, 6 n-hexanol-water binary mixtures, and 6 benzyl alcohol-n-hexanol-water ternary mixtures.

The Redlich and Kister model [26] is one of the most used models for representing the thermodynamic properties of excess:

(1) $$\Delta Y=x\cdot \left(1-x\right)\underset{i}{\sum }A_{i}\cdot {\left(2x-1\right)}^{i}$$

where *x* is the mole fractions and *A_i_* is the coefficients in the polynomial equation.

The number of parameters that must be used to represent the experimental data depends on the distribution of the data and is directly related to the complexity of the process, the quality of the data and also the number of available experimental data. Thus, for binary mixtures, the equation has the following form:

(2) $$\Delta Y=x_{1}x_{2}\left[A_{12}+B_{12}\cdot \left(x_{1}-x_{2}\right)+C_{12}\cdot {\left(x_{1}-x_{2}\right)}^{2}+\cdots \right]$$

where *x*_1_, *x*_2_ is the mole fractions and *A*_12_, *B*_12_, and *C*_12_ are the coefficients in the polynomial equation.

Considering that for binary mixtures, the complementarity of the mole fractions is verified, then $x_{1}-x_{2}=2x_{1}-1$, and knowing that $A_{12}=A_{0},B_{12}=A_{1},\;C_{12}=A_{2},\ldots ,$ etc., Equation (2) will have the following form:

(3) $$\Delta \eta =x_{1}\left(1-x_{1}\right)\overset{n-1}{\underset{i=0}{\sum }}A_{i}{\left(2x_{1}-1\right)}^{i}$$

in which the coefficient *A_i_* is obtained through a least squares regression procedure. The number of coefficients used in Equation (3) depends on the criterion chosen by the researcher, using evaluations for this, including the number of experimental data, their distribution, the purpose for which they are obtained, etc.

In the case of ternary systems, the Redlich and Kister model takes into account the relation (3), which allows the calculation of deviations from the ideal behavior for binary mixtures and Equation (4) for ternary mixtures [26].

(4) $$\begin{array}{c} \Delta Y_{1,2,3}=\Delta Y_{1,2}+\Delta Y_{1,3}+\Delta Y_{2,3}\\ \;\;\;\;+x_{1}\cdot x_{2}\cdot x_{3}\left[A_{0,123}+A_{1,123}\cdot x_{1}\cdot \left(x_{2}-x_{3}\right)+A_{2,123}\cdot x_{1}^{2}\cdot {\left(x_{2}-x_{3}\right)}^{2}+A_{3,123}\cdot x_{1}^{3}\cdot {\left(x_{2}-x_{3}\right)}^{3}\right] \end{array}$$

where Δ*Y*_1,2_, Δ*Y*_1,3_, and Δ*Y*_2,3_ are the deviations for binary mixtures, *x*_1_, *x*_2_, and *x*_3_ are the mole fractions, and *A*_0,123_, *A*_1,123_, *A*_2,123_, and *A*_3,123_ are the coefficients in the polynomial equation for ternary mixtures.

The relation between viscosity deviation, composition, normalized temperature, and refractive index was evaluated using multiple linear regression (MLR) and artificial neural networks.

The viscosity deviation calculated based on the experimental determinations of the viscosity of pure compounds and binary and ternary mixtures was modeled by statistical processing of the experimental data with the multiple linear regression method (MLR). The use of Sigmaplot 11 software (Systat Software Inc., San Jose, CA, USA) allowed establishment of the dependence between the viscosity deviation (dependent variable) and the composition (X_1_ şi X_2_), the normalized temperature (T/273.15), and the refractive index (independent variables).

Modeling with the help of neural networks was carried out with the Neurosolutions commercial simulator produced by the NeuroDimension company (NeuroDimension Inc., Boston, MA, USA). This simulator has the advantage of providing users with a set of neuron models, data set interaction models, and training algorithm models. They are easy to use and have a visual representation that allows the user to easily build their neural network structure. Through a graphic interface, the software provides elementary blocks that, when combined, can generate various neural architectures. In this study, neural models of multilayer perceptrons were built. The type of transfer function selected was TanhAxon and learning rule momentum.

**Scheme 1:**
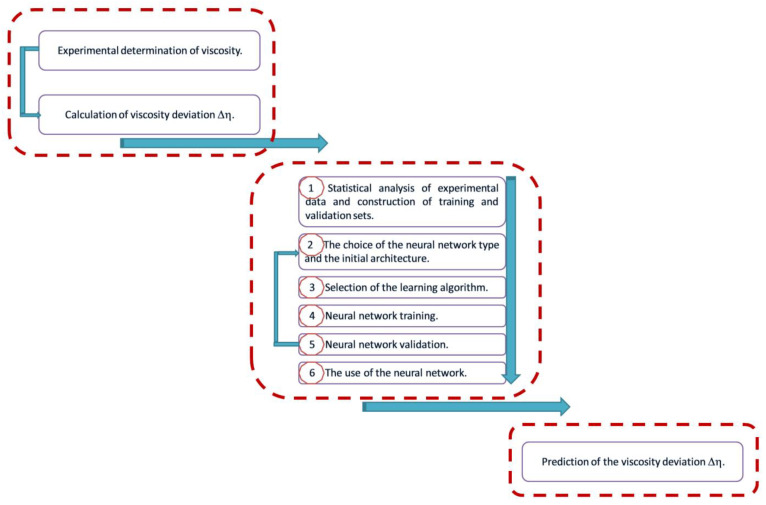
Viscosity deviation modeling methodology.

The methodology for modeling the viscosity deviation based on experimental determinations of the refractive index and viscosity at different temperatures and mole fractions of n-hexanol and benzyl alcohol involves following the steps shown in Scheme 1.

The performance evaluation of the built models was carried out by calculating the standard deviation with the following relationship:

(5) $$\sigma =\sqrt{\overset{k}{\underset{i=1}{\sum }}{\left[\Delta \eta_{experimental}-\Delta \eta_{model}\right]}^{2}/\left(n-p\right)}$$

where *n* represents the number of experimental data and *p* is the number of parameters.

The performances of the built neural models were evaluated based on the mean squared error (MSE) and the correlation coefficient (r^2^):

(6) $$\mathrm{MSE}=\left(\sum_{j=1}^{P}\sum_{i=1}^{N}{\left(D_{\mathrm{ij}}-O_{\mathrm{ij}}\right)}^{2}\right)/\left(N\cdot P\right)$$

where N is the number of data, P is the number of output quantities (in this case, P = 1), O_ij_ is the output value for element i with element j processing, and D_ij_ is the desired output for i with element j processing;

(7) $$r^{2}=\frac{\sum \left(O_{\mathrm{expi}}-{\bar{O}}_{\exp}\right)\cdot \left(O_{\mathrm{neti}}-{\bar{O}}_{\mathrm{net}}\right)}{\sqrt{\sum {\left(O_{\mathrm{expi}}-O_{\exp}\right)}^{2}\cdot {\left(O_{\mathrm{neti}}-{\bar{O}}_{\mathrm{net}}\right)}^{2}}}$$

where O are the values of the output data, respectively, and exp and net denote the experimental values and those obtained from neural models.

Other regression algorithms were also used for comparison: nearest neighbor (NN), k-nearest neighbor (kNN), K*, support vector regression (SVR), and random forest. For the kNN algorithm that searches for the most suitable training examples in the feature space and then uses their average as a prediction, k = 3 or 10 neighbors and the inverse distance function: w_i_ = 1/d_i_ were used, and in the NN algorithm, k = 1 was considered. For the K* algorithm, the global parameter gb = 10, 20, or 50 was considered. This parameter can be considered a sphere of influence that implicitly specifies how many neighbors are significant [27]. Support Vector Regression (SVR) is a method that seeks to minimize error by finding output values that lie within a given range. Three types of kernels were used: polynomial, Radial Basis Function (RBF), and Pearson Universal Kernel (PUK); and cost parameters C = 100 or 10,000. This cost parameter controls the strictness of the objective function that is optimized by the algorithm. If a greater value is used for C, there will be a smaller margin of error, but it results in a lower generalizability [27]. The random forest algorithm involved 100 or 1000 decision trees. Each decision tree calculates an output value, and the forest ensemble calculates the average of these individual values [27].

## 3. Results and Discussions

### 3.1. Experimental Determination of Thermodynamic Properties for Binary and Ternary Systems

**Figure 1:**
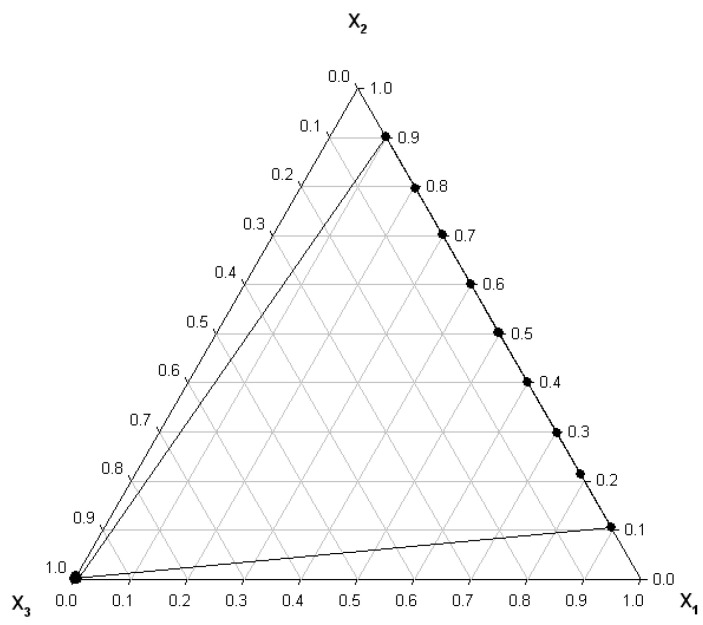
Ternary graph for the system: benzyl alcohol (1)—n-hexanol (2)—water (3).

The ternary graph presented in Figure 1 indicates that experimental determinations were made for the entire range of variation of mole fractions for the benzyl alcohol (1)—n-hexanol (2) binary system and in the field of dilute solutions for the benzyl alcohol (1)—water (3) and n-hexanol (2)—water (3) binary systems and for the benzyl alcohol (1)—n-hexanol (2)—water (3) ternary system.

The viscosity values experimentally measured at different temperatures for pure compounds are presented in Table 1, and those for binary and ternary mixtures are presented in Table 2.

Table 1 reports the experimental data from this study to other data provided by the specialized literature. Close values with those reported by other researchers at the same temperature are found for the viscosities of the pure compounds.

The viscosity of the binary and ternary mixtures, according to the results presented in Table 2, decreases as the temperature increases from 293.15 to 323.15 K. In the case of the benzyl alcohol (1)—water (2) binary system, the viscosity increases slightly with the increase in the molar fraction of benzyl alcohol at the same temperature. For the benzyl alcohol (1)—n-hexanol (2) binary system, the viscosity decreases slightly up to molar fractions X_1_ = 0.3, and then, due to the intensification of the interactions between the components, the viscosity increases with the increase of the molar fraction of benzyl alcohol at the same temperature. In this binary system, the predominant interactions between the molecules of benzyl alcohol and n-hexanol are of the type (O … H—O), but there can also be weaker dipole-dipole interactions [34]. For the n-hexanol-water binary system and the ternary system, the viscosity varies less with composition at the same temperature.

**Figure 2:**
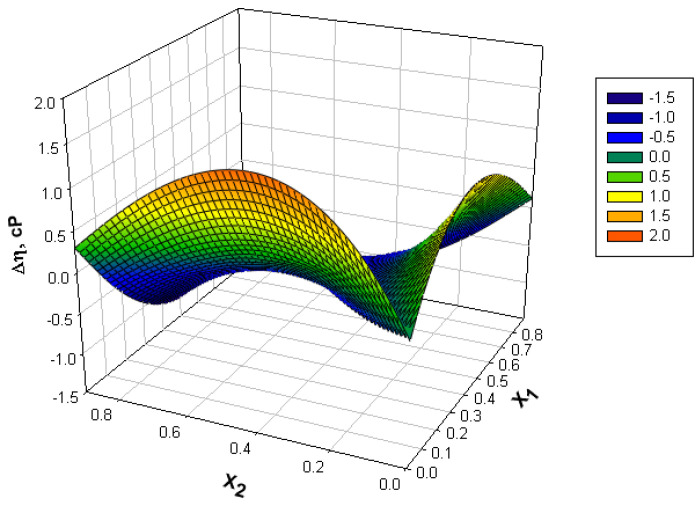
Variation of viscosity deviation (Δ*η*) with mole fractions at different temperatures.

The deviation from the ideal behavior is highlighted by the viscosity deviation (Δ*η*) (Figure 2), defined by relation (8):

(8) $$\Delta \eta =\eta -\overset{n}{\underset{i=1}{\sum }}x_{i}\eta_{i}$$

where *n* varies from 1 to 3, *η* is the viscosity of binary/ternary systems, *η_i_* is the viscosity of pure compounds, and *x_i_* is the mole fractions.

**Figure 3:**
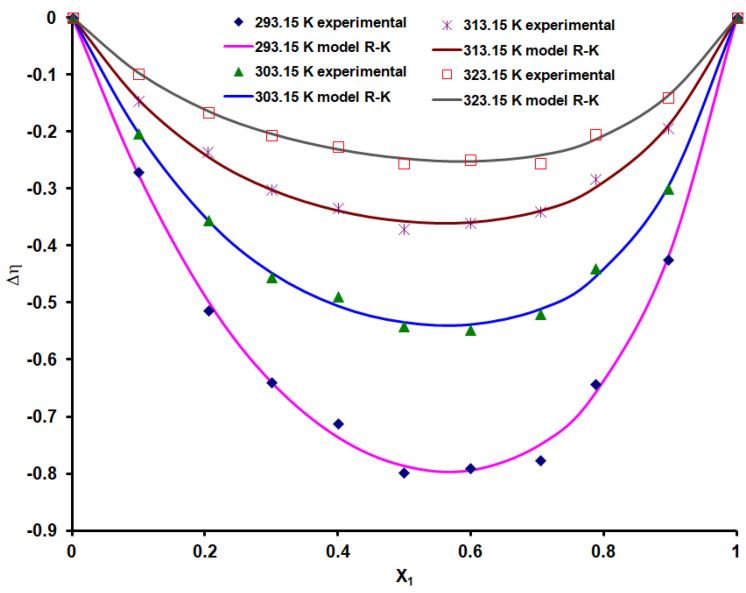
Viscosity deviation values for the benzyl alcohol (1)—n-hexanol (2) binary system.

According to the values obtained for the viscosity deviation graphically represented in Figure 2, the binary and ternary systems containing water generally give positive deviations, and the benzyl alcohol (1), n-hexanol (2) binary system gives negative deviations from the ideal behavior. In systems containing water, positive viscosity deviations can be attributed to specific interactions between different molecules in the mixture [35]. In the benzyl alcohol (1)—n-hexanol (2) binary system, negative values appear due to the disruption or breaking of the associative bonds of the molecules [9]. For this binary system (Figure 3) lower and lower values of the viscosity deviation are found as the temperature increases and a minimum is observed near the equimolar composition.

**Figure 4:**
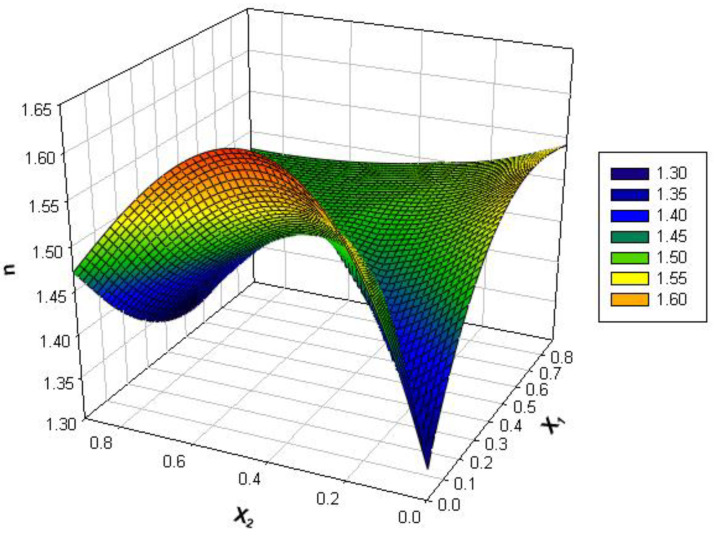
Variation of refractive index with mole fractions at different temperatures.

The refractive index is determined experimentally quite easily but also with a fairly good measurement precision and with a small consumption of materials. According to specialized literature, models can be built that correlate this parameter with other properties that are much more difficult to measure, such as density, surface tension, or viscosity [18,19,36]. For the binary and ternary systems [36] analyzed in this study, the refractive index (Figure 4) varies depending on composition and temperature between 1.3 and 1.6.

### 3.2. Statistical Analysis of Experimental Data and Construction of Training and Validation Sets

The statistical processing of the experimental data was carried out with the specialized program SigmaPlot 11.00. The program allowed the determination of mean values, standard deviation, and standard mean error. Also, the confidence interval of the mean, the amplitude, the minimum and maximum values, the median, and the distribution interval of 25% and 75% of the data were established. The evaluation of the normal distribution of the data was carried out by the Skewness and Kurtosis tests, the Kolmogorov–Smirnov test, the Shapiro–Wilk test, and by calculating the sum of the data and the sum of squares [27]. According to the data presented in Table 3, there are no missing data. The analysis of the distribution of the experimental data (Skewnss) reveals a positive asymmetry for the mole fractions, the normalized temperature, and the refractive index and a negative one for the viscosity deviation. The Kolmogorov–Smirnov normality test was also applied, which quantifies the degree of overlap between the cumulative distribution of the analyzed variables and the cumulative distribution of the variable that follows the shape of the Gaussian curve. The obtained values indicate a normal distribution of the experimental data and the fact that they are statistically significant (*p* < 0.001), an aspect confirmed by the results obtained with the Shapiro–Wilk normality test.

### 3.3. Modeling the Thermodynamic Properties of Excess with the Redlich and Kister Model

To model the viscosity deviation, the applicability of the Redlich and Kister (R-K) polynomial model was verified, which correlates this thermodynamic quantity with the composition (relations 3 and 4). The coefficients A_0_...A_3_ and the standard deviation for these models are presented in Table 4. Figure 3 compares the values calculated based on the experimental determinations for the viscosity deviation and those obtained from the Redlich and Kister (R-K) model for the benzyl alcohol (1)—n-hexanol (2) binary system. It is found that the experimental values are very close to those calculated with the R-K model, and the standard deviations are between 0.0069 and 0.0161.

If we analyze the standard deviations shown in Table 4, we find very good performance for this type of model for all the evaluated systems, but its disadvantage is the fact that it requires one equation for each temperature, a lot of calculations to obtain them, and respectively, a longer experimental data processing time.

### 3.4. Multiple Linear Regression (MLR) Method

In previous studies [18,19,36], it was demonstrated that good results were obtained for the correlation of excess thermodynamic properties with normalized temperature, refractive index, and mole fractions, with the help of models obtained by the multiple linear regression method.

**Figure 5:**
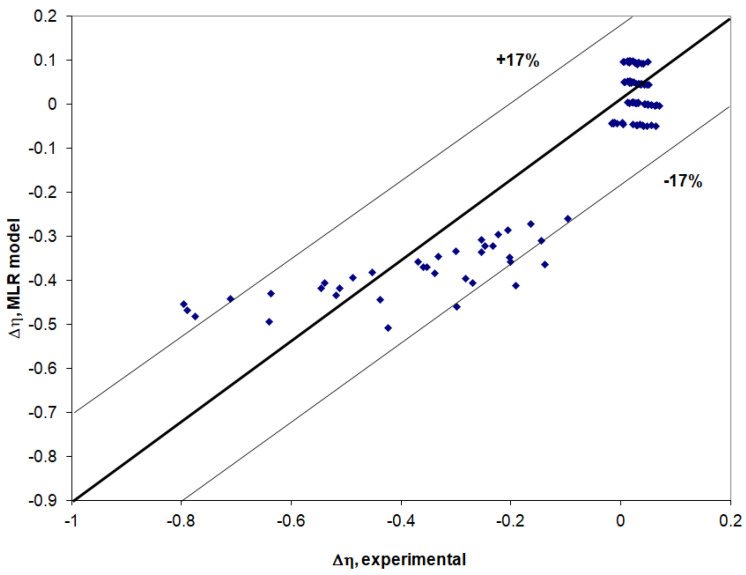
Viscosity deviation calculated with the (MLR) proposed model (Equation (9)) compared to experimental values.

In this study, for modeling the viscosity deviation, an MLR model was built for which the standard deviation is 0.099, the correlation coefficient is 0.898, and most of the values obtained are located within a confidence interval of ±17% (Figure 5). The proposed MLR model has the following mathematical expression:

Δη = −0.301 − (0.315·X_1_) − (0.283·X_2_) + (1.259 ⸱ T/273.15) − (0.816·n) (9)

### 3.5. Modeling with Neural Networks

To build the training and validation data sets, the mixing of the 108 available data series was used. For this purpose, their processing in Excel was used by generating random numbers for the experimental data, which were then ordered in ascending or descending order, thus resulting in a good mixing of them. To generate the random numbers, the following expression “=INT(n*RAND()+1)” was used, where n is a number equal to or greater than the number of experimental data. A total of 87% of the available data were used for training and 13% were used in the validation stage.

After mixing the 108 available experimental data, according to the mentioned algorithm, 93 data were used in the training stage. Neural models were built with four neurons in the input layer, one or two layers of hidden neurons, and one output. To avoid overtraining the neural models, the variation of the mean squared error (MSE) with the increase in the number of epochs was followed, and it was determined that the optimal number of training epochs is 60,000. For all the neural models, this result was taken into account, and their performances are presented comparatively in Table 5.

**Figure 6:**
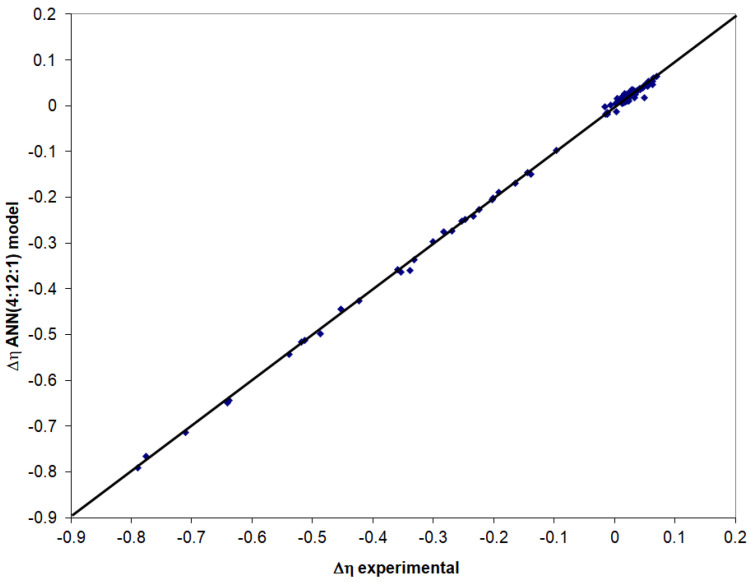
Evaluation of the ANN(4:12:1) model in the testing stage.

The neural model containing 4 neurons in the input layer, 12 neurons in the hidden layer, and one neuron in the output layer led to the best performance in the training stage. Figure 6 compares the experimental data with those provided by the ANN(4:12:1) model in the testing stage.

The calculated standard deviation for the ANN(4:12:1) model in the testing stage with relation (5) is 0.0067, and the correlation coefficient is 0.999.

**Figure 7:**
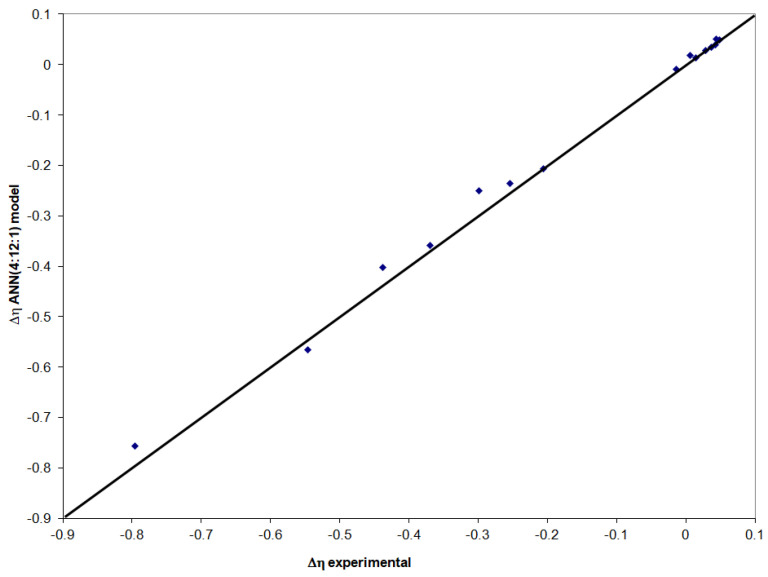
Evaluation of the ANN(4:12:1) model in the validation stage.

For the neural model validation stage, 15 series of experimental data were used. According to the representation in Figure 7, very good results are obtained, and the values calculated with the ANN(4:12:1) model for the viscosity deviation are very close to the experimental values. The standard deviation in this step of neural model validation is 0.0226, and the correlation coefficient is 0.996.

If we compare the results obtained with the ANN(4:12:1) model, with those given by the previously presented MLR model, we see the clearly superior performance of the neural model. The standard deviation for the neural model is several times smaller than for the MLR model, and the correlation coefficient is higher than 0.99 for ANN compared to 0.898 for MLR.

### 3.6. Modeling with Regression Methods

On the same database, modeling was done with different regression methods: nearest neighbor (NN), k-nearest neighbor (kNN), K*, support vector regression (SVR), and random forest. The results of the regression with different algorithms and combinations of parameter values are presented in Table 6. In this table, both the results obtained in the training stage and in the cross-validation are presented.

Similar results to neural models were obtained in the cross-validation stage with two of the SVR models, respectively with radial basis function (RBF) kernels with cost parameter C = 100 and Pearson universal kernel (PUK) with C = 10,000.

## 4. Conclusions

With high-performance equipment, i.e., the Physica MCR 501 modular rheometer, equipped with a CC27 concentric cylinder measuring system, at a shear rate of 10 s^−1^ and at temperatures of 293.15, 303.15, 313.15, and 323.15 K, the viscosities for pure compounds and binary and ternary mixtures of benzyl alcohol, n-hexanol, water, were determined.

The viscosity deviation was calculated, which has negative values for the benzyl alcohol (1)-n-hexanol (2) binary system and generally positive values for the binary and ternary systems containing water. A database was created that was statistically processed and with the help of which models were built that correlate viscosity deviation with temperature, composition, and refractive index.

Very good results were obtained with the ANN(4:12:1) model. The viscosity deviation values are very close to the experimental ones; the standard deviation in the test stage is 0.0067, and in the validation stage, it is 0.0226. The correlation coefficients are 0.999 in the testing stage and 0.996 in the validation stage. In the cross-validation stage, close results were also obtained with two regression models, namely SVR (C = 100, RBF γ = 1) and SVR (C = 10,000, PUK).

The results obtained with the MLR model are worse than those obtained with the neural model and the Redlich and Kister type model. The standard deviation is 0.099, and the correlation coefficient is 0.898 for the MLR model. Its advantage over those of Redlich and Kister type polynomials is that a single equation is obtained that correlates the viscosity deviation with temperature, composition, and refractive index.

## Figures and Tables

**Table 1:** Viscosity of pure compounds (n-hexanol, benzyl alcohol, water) determined experimentally.

Substantance	Temperature, K	Viscosity η, cP	
		Experimental	Literature
n-hexanol	293.15	5.258	5.35 ^a^; 5.362 ^c^
	303.15	3.890	3.84 ^b^; 3.93 ^c^ 3.829 ^d^
	313.15	2.920	2.90 ^b^; 2.95 ^c^ 2.879 ^d^
	323.15	2.240	2.23 ^b^; 2.259 ^c^ 2.215 ^d^
benzyl alcohol	293.15	6.430	6.287 ^h^ ; 6.421 ^k^
	303.15	4.735	4.1352 ^e^; 4.4255 ^f^; 4.689 ^g^; 4.579 ^h^;4.515 ^l^
	313.15	3.535	3.2061 ^e^; 3.531 ^g^ 3.335 ^h^
	323.15	2.740	2.747 ^g^; 2.545 ^h^
water	293.15	0.989	1.016 ^i^; 1.0050 ^j^
	303.15	0.789	0.7972 ^i^; 0.8007 ^j^
	313.15	0.645	0.6527 ^i^; 0.6560 ^j^
	323.15	0.548	0.5465 ^i^

Standard uncertainties u are u(T) = ±0.01 K and u(η) = ±0.02 cP; ^a^ [4], ^b^ [28], ^c^ [9], ^d^ [10], ^e^ [29], ^f^ [30], ^g^ [31], ^h^ [14], ^i^ [32], ^j^ [33], ^k^ [11], ^l^ [12].

**Table 2:** Viscosity of binary and ternary mixtures of: benzyl alcohol (1), n-hexanol (2) and water (3).

System	X_1_	X_2_	η, cP			
Temperature, K			293.15	303.15	313.15	323.15
binary benzyl alcohol—water	0.0012	0	0.981	0.806	0.658	0.548
	0.0023	0	0.997	0.815	0.664	0.553
	0.0031	0	1.020	0.829	0.676	0.564
	0.0040	0	1.030	0.840	0.683	0.569
	0.0051	0	1.050	0.850	0.690	0.572
	0.0059	0	1.060	0.861	0.699	0.576
binary benzyl alcohol—n-hexanol	0.0989	0.9011	5.103	3.775	2.845	2.198
	0.2037	0.7963	4.983	3.710	2.820	2.183
	0.2991	0.7009	4.970	3.690	2.813	2.190
	0.3997	0.6003	5.015	3.740	2.843	2.223
	0.4978	0.5022	5.045	3.770	2.865	2.243
	0.5985	0.4015	5.170	3.848	2.938	2.300
	0.7036	0.2964	5.308	3.963	3.023	2.348
	0.7869	0.2131	5.540	4.113	3.130	2.440
	0.8958	0.1042	5.885	4.343	3.288	2.560
binary n-hexanol—water	0	0.0011	0.969	0.794	0.649	0.540
	0	0.0009	0.971	0.790	0.646	0.538
	0	0.0007	0.986	0.809	0.664	0.556
	0	0.0005	0.971	0.799	0.657	0.550
	0	0.0003	0.970	0.798	0.654	0.548
	0	0.0001	0.967	0.796	0.651	0.545
ternary benzyl alcohol—n-hexanol-water	0.0003	0.0033	1.030	0.835	0.683	0.572
	0.0002	0.0013	0.989	0.808	0.663	0.584
	0.0003	0.0059	1.070	0.864	0.703	0.585
	0.0003	0.0053	1.060	0.857	0.699	0.581
	0.0004	0.0043	1.040	0.846	0.688	0.572
	0.0006	0.0038	1.030	0.833	0.681	0.567

Standard uncertainties u are u(T) = ±0.01 K and u(η) = ±0.02 cP.

**Table 3:** Statistical description of the database.

**No.** **Col**	**Size**	**Missing**	**Mean**	**Std Dev**	**Std. Error**	**C.I. of Mean**	**Range**	**Max**	**Min**	**Median**
1	108	0	0.167	0.278	0.0268	0.0531	0.896	0.896	0.000	0.0022
2	108	0	0.168	0.280	0.0269	0.0533	0.901	0.901	0.000	0.0033
3	108	0	1.128	0.041	0.0039	0.0078	0.110	1.183	1.073	1.128
4	108	0	1.384	0.066	0.0063	0.0126	0.192	1.527	1.334	1.341
5	108	0	−0.111	0.224	0.0216	0.0428	0.866	0.0687	−0.797	0.0134
**No.** **Col**	**25** **%**	**75** **%**	**Ske-Wnss**	**Kur-Tosis**	**K-S Dist.**	**K-S** **Prob.**	**Shapiro–Wilk**	**Shapiro–Wilk** **Prob.**	**Sum**	**Sum of Squares**
1	0.000160	0.299	1.467	0.717	0.385	<0.001	0.649	<0.001	18.031	11.293
2	0.000146	0.296	1.466	0.725	0.386	<0.001	0.651	<0.001	18.174	11.421
3	1.092	1.165	0.000	−1.367	0.172	<0.001	0.856	<0.001	121.839	137.36
4	1.338	1.447	0.942	−0.887	0.390	<0.001	0.703	<0.001	149.443	207.25
5	−0.230	0.0307	−1.539	1.420	0.328	<0.001	0.729	<0.001	−11.964	6.707

Col 1-x_1_, Col 2-x_2_, Col 3—normalized temperature, Col 4—refractive index, Col 5—viscosity deviation.

**Table 4:** Parameters and standard deviation for the Redlich and Kister model.

System	Temperature, K	A3	A2	A1	A0	σ
benzyl alcohol—water	293.15	2.482 × 10^7^	7.366 × 10^7^	7.366 × 10^7^	2.402 × 10^7^	0.0022
	303.15	−3.616 × 10^7^	−1.075 × 10^7^	−1.066 × 10^8^	−3.523 × 10^7^	0.0024
	313.15	−2.012 × 10^7^	−5.986 × 10^7^	−5.936 × 10^7^	−1.962 × 10^7^	0.0021
	323.15	−1.608 × 10^7^	−4.788 × 10^7^	−4.752 × 10^7^	−1.572 × 10^7^	0.0021
benzyl alcohol—n-hexanol	293.15	−0.542	−1.049	−0.577	−3.154	0.0161
	303.15	−0.426	−0.940	−0.315	−2.139	0.0098
	313.15	−0.107	−0.627	−0.207	−1.425	0.0080
	323.15	−0.047	−0.465	−0.215	−0.994	0.0069
n-hexanol—water	293.15	3.743 × 10^10^	1.121 × 10^11^	1.118 × 10^11^	3.723 × 10^10^	0.0039
	303.15	−4.526 × 10^10^	−1.355 × 10^11^	−1.353 × 10^11^	−4.505 × 10^10^	0.0068
	313.15	−1.305 × 10^10^	−3.909 × 10^10^	−3.903 × 10^10^	−1.299 × 10^10^	0.0025
	323.15	−1.269 × 10^10^	−3.803 × 10^10^	−3.797 × 10^10^	−1.263 × 10^10^	0.0047
benzyl alcohol—n-hexanol-water	293.15	−4.492 × 10^15^	−5.523 × 10^12^	−2.059 × 10^9^	−2.023 × 10^5^	0.0039
	303.15	6.036 × 10^15^	7.833 × 10^12^	3.312 × 10^9^	4.866 × 10^5^	0.0059
	313.15	4.092 × 10^15^	5.366 × 10^12^	2.297 × 10^9^	3.417 × 10^5^	0.0047
	323.15	1.627 × 10^16^	2.089 × 10^13^	8.557 × 10^9^	1.129 × 10^6^	0.0126

1-benzyl alcohol, 2-n-hexanol, 3-water; for the ternary system A_0,123_…A_3,123_ (Equation (4)).

**Table 5:** The structure of neural models and the performances obtained in the training stage.

No.	Topology	MSE	r^2^	The Duration of the Training Process (min)
1.	ANN(4:4:1)	0.000422	0.998279	1.38
2.	ANN(4:8:1)	0.000232	0.999430	1.55
3.	ANN(4:12:1)	0.000204	0.999512	1.37
4.	ANN(4:16:1)	0.000248	0.999391	1.29
5.	ANN(4:20:1)	0.000239	0.999412	2.11
6.	ANN(4:8:4:1)	0.000245	0.999426	2.19
7.	ANN(4:12:4:1)	0.000227	0.999442	2.37

**Table 6:** The results obtained with several regression algorithms.

Algorithm	Training	Cross-Validation
kNN (k = 3, w_i_ = 1/d_i_)	0.9999	0.9715
kNN (k = 10, w_i_ = 1/d_i_)	0.9996	0.9616
NN (k = 1)	1	0.9629
K* (gb = 10)	0.9996	0.9718
K* (gb = 20)	0.9981	0.9711
K* (gb = 50)	0.9860	0.9549
SVR (C = 10,000, PUK)	0.9997	0.9978
SVR (C = 100, poly d = 2)	0.9880	0.9785
SVR (C = 100, RBF γ = 1)	0.9994	0.9971
Random Forest (100 trees)	0.9981	0.9819
Random Forest (1000 trees)	0.9982	0.9842
